# Memantine Attenuates Delayed Vasospasm after Experimental Subarachnoid Hemorrhage via Modulating Endothelial Nitric Oxide Synthase

**DOI:** 10.3390/ijms160614171

**Published:** 2015-06-23

**Authors:** Chih-Yuan Huang, Liang-Chao Wang, Yan-Shen Shan, Chia-Hsin Pan, Kuen-Jer Tsai

**Affiliations:** 1 Institute of Clinical Medicine, College of Medicine, National Cheng Kung University, Tainan 704, Taiwan; E-Mails: gordon168999@yahoo.com.tw (C.-Y.H.); liangcha@mail.ncku.edu.tw (L.-C.W.); ysshan@mail.ncku.edu.tw (Y.-S.S.); proie628@yahoo.com.tw (C.-H.P.); 2 Department of Surgery, National Cheng Kung University Hospital, College of Medicine, National Cheng Kung University, Tainan 704, Taiwan; 3 Center of Clinical Medicine, National Cheng Kung University Hospital, College of Medicine, National Cheng Kung University, Tainan 704, Taiwan

**Keywords:** memantine, nitric oxide synthase, subarachnoid hemorrhage, vasospasm

## Abstract

Delayed cerebral vasospasm is an important pathological feature of subarachnoid hemorrhage (SAH). The cause of vasospasm is multifactorial. Impairs nitric oxide availability and endothelial nitric oxide synthase (eNOS) dysfunction has been reported to underlie vasospasm. Memantine, a low-affinity uncompetitive *N*-methyl-d-aspartate (NMDA) blocker has been proven to reduce early brain injury after SAH. This study investigated the effect of memantine on attenuation of vasospasm and restoring eNOS functionality. Male Sprague-Dawley rats weighing 350–450 g were randomly divided into three weight-matched groups, sham surgery, SAH + vehicle, and SAH + memantine groups. The effects of memantine on SAH were evaluated by assessing the severity of vasospasm and the expression of eNOS. Memantine effectively ameliorated cerebral vasospasm by restoring eNOS functionality. Memantine can prevent vasospasm in experimental SAH. Treatment strategies may help combat SAH-induced vasospasm in the future.

## 1. Introduction

Subarachnoid hemorrhage (SAH) accounts for 5%–10% of all cerebrovascular accidents and is a major cause of disability and death in humans [1]. Approximately 1 in every 10,000 population suffers aneurysm rupture every year [2]. A major complication of aneurysmal SAH is symptomatic vasospasm, which usually occurs 4–12 days after SAH [1]. Cerebral vasospasm is the vasoconstriction of the conducting arteries of the circle of Willis, leading to secondary cerebral ischemia. Its pathogenesis is complex and still cannot be elucidated fully.

Nitric oxide (NO), a diffusible factors and a powerful dilator, causes a number of beneficial effects such as antithrombotic effects, prevention of excess platelet adhesion and aggregation, maintenance of vascular tone and regulation of cerebral blood flow [3,4]. It activates soluble guanylyl cyclase and up-regulates 3ʹ–5ʹ cGMP and dilates the arteries in response to metabolic demand and shear stress. NO is produced enzymatically from l-arginine by three main nitric oxide synthase (NOS) isozymes, endothelial, neuronal, and inducible NOS, or in a nonenzymatic fashion via nitrate–nitrite reduction–oxidation reactions [5].

Several studies report that the nitric oxide pathway is impaired after SAH, resulting in delayed vasospasm [6]. Oxyhemoglobin released from the subarachnoid clot scavenges NO and destroys neuronal NOS-containing neurons in the adventitia [7], which deprives the arteries of NO. SAH also causes endothelial NOS (eNOS) dysfunction as it leads to increased levels of an endogenous NOS inhibitor, asymmetric dimethylarginine [7]. Another possible mechanism of eNOS dysfunction is uncoupling of eNOS. It has been reported that SAH-induced oxidative stress correlates with eNOS homodimeric uncoupling, which exacerbates oxidative stress and further enhances NO depletion [8]. This finding is also supported by the results, which showed that eNOS knockout significantly alleviates vasospasm and reduces superoxide production after SAH [9].

Memantine, a low-affinity uncompetitive NMDA blocker, has been shown to be effective in preventing neuronal damage after models of neurological injury [10], including SAH [11]. It has been reported memantine attenuated production of reactive oxygen species after SAH by diminishing the impairment of neurovascular units and preserving the integrity brain-blood barrier [11]. The present study examined the therapeutic effects of memantine in SAH-induced endothelial dysfunction and delayed vasospasm.

## 2. Results

### 2.1. Effect of Memantine on Attenuation of Delayed Vasospasm

**Figure 1:**
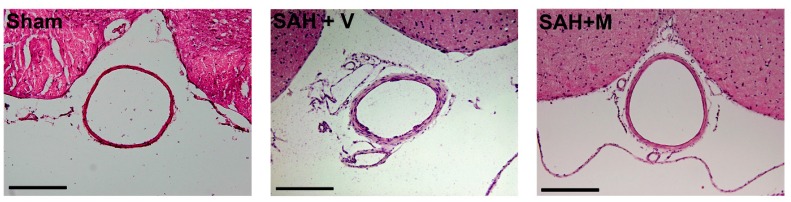
Subarachnoid hemorrhage (SAH) induced delayed vasospasm. Photographs of representative hematoxylin-eosin-stained cross-sections of the basilar artery demonstrated decreased lumen area of the basilar artery in vehicle-treated SAH animals and memantine treatment ameliorated the vasospasms (Scale bars = 200 μm).

**Figure 2:**
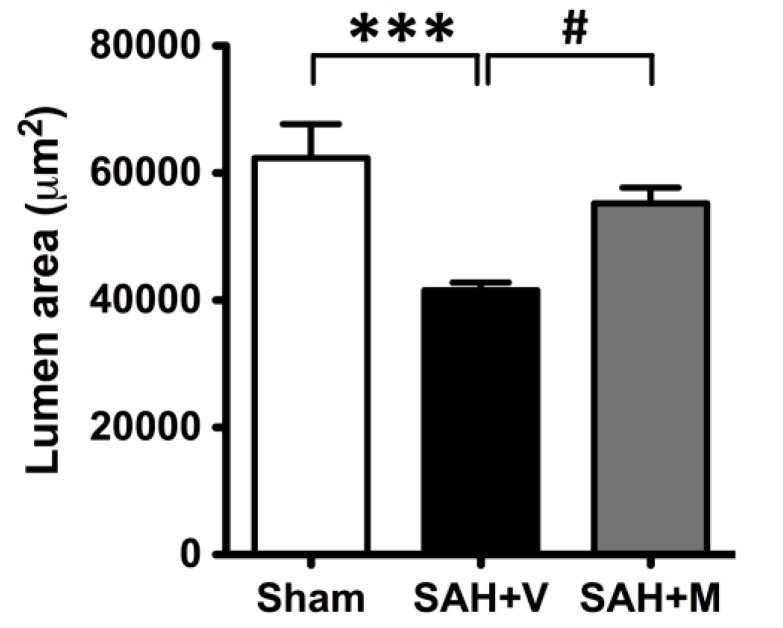
Memantine attenuated the delayed vasospasm. Measurements of lumen area of the basilar artery were shown for each group under corresponding bar graphs. The SAH animals showed statistically significant reduction of area than the sham-operated group. Memantine significantly recovered the area, indicating attenuation of the vasospasm. All data were mean ± SEM (*n* = 8 per group). *** *p* < 0.001 *vs.* sham, # *p* < 0.05 *vs.* SAH + V.

Photographs of representative hematoxylin-eosin-stained cross-sections of the basilar artery demonstrated decreased lumen area of the basilar artery due to vasospasms in vehicle-treated SAH animals and that memantine treatment ameliorated the vasospasms (Figure 1). To examine the effect of memantine on attenuation of delayed vasospasm after SAH, the lumen area of the basilar artery was measured for each group as shown in the corresponding bar graphs (Figure 2). The lumen areas of sham, SAH + vehicle (SAH + V) and SAH + memantine (SAH + M) group were 62,366.9 ± 2390.1, 41,586.4 ± 458.0 and 55,261.9 ± 1099.0 μm^2^, respectively. Basilar arteries (BA) of rats in the sham group had significantly larger lumen area, while the SAH + V group exhibited prominent BA vasospasm and decreased lumen. Memantine recovered the lumen area (*p* < 0.001) (Figure 2), indicating attenuation of the vasospasm.

### 2.2. Memantine Attenuated Vasospasm via Mediating Endothelial Nitric Oxide Synthase (eNOS) Functionality

**Figure 3:**
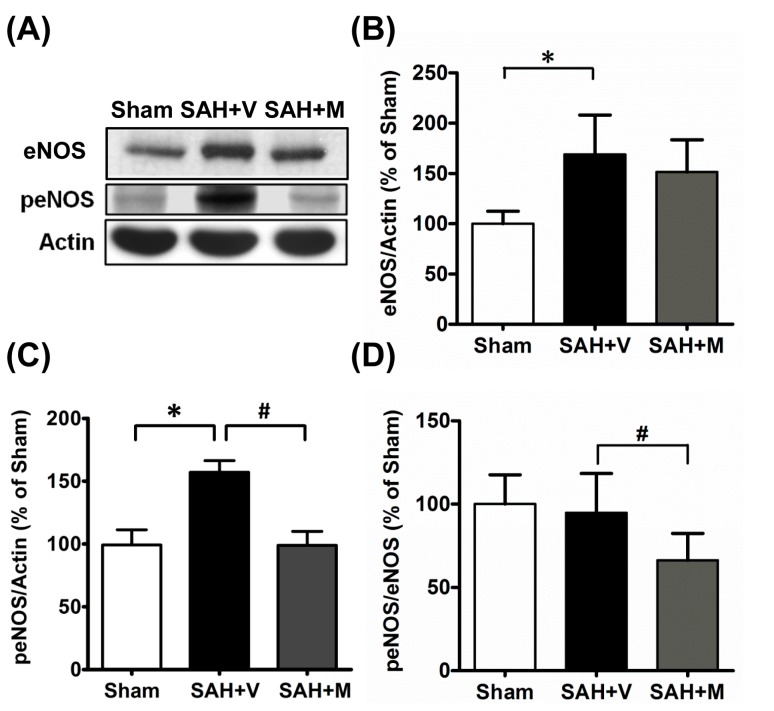
Memantine diminished the expression of phosphorylated endothelial nitric oxide synthase. (**A**) Representative Western blots probed for endothelial nitric oxide synthase (eNOS) and phosphorylated eNOS in the brain tissue; Corresponding bar graphs showed statistically significant up-regulation of eNOS (**B**) and phosphorylated eNOS (**C**) in SAH + V animals compared to the sham-operated group; and (**D**) Treatment with memantine significantly restored phosphorylated eNOS level. * *p* < 0.05 *vs.* sham, # *p* < 0.05 *vs.* SAH + V.

To investigate the underlying mechanism by which memantine attenuated the vasospasm after SAH, the expressions of eNOS, phosphorylated eNOS (peNOS) and peNOS/eNOS were examined. The SAH substantially increased the expression peNOS compared to that observed in the sham group (*p* < 0.05). Memantine treatment significantly down-regulated peNOS expression in the SAH+M group compared to the SAH + V group (*p* < 0.05) (Figure 3). Thus, memantine might attenuate vasospasm after SAH via mediating eNOS functionality.

## 3. Discussion

The features of the endovascular perforation SAH model used in this study closely resemble those of aneurysmal SAH in humans, including the high mortality rate, decreased cerebral blood flow (CBF) and delayed vasospasm [12,13,14]. Memantine is reported as an open-channel NMDA blocker with a faster off-rate than many NMDA antagonists [15,16]. This favorable pharmokinetic reaction with NMDA receptor may be responsible for its high index of therapeutic safety [10]. Therefore, memantine has been used as a treatment of dementia and Parkinson’s disease for several years without serious side effects and it has been demonstrated that memantine could reduce SAH-induced early brain injury [11]. This study highlights that memantine specifically attenuates SAH-induced delayed vasospasm via mediating eNOS functionality.

### 3.1. Memantine Prevents Neurovascular Damage and Development of Vasospasm

SAH-induced global ischemia causes excessive production of glutamate, which can over-activate NMDAR [11]. Previous reports have demonstrated the presence of NMDAR in cerebral endothelial cells [17,18]. Therefore, the activation of NMDAR and subsequent induction of oxidative/nitrosative stress lead to the damage of neurons, glias, as well as endothelial cells [11,19,20]. Neuronal and endothelial damage result in the disruption of neurovascular units, increased permeability, and/or edema formation in SAH [21], which in turn impair cerebral circulation in the acute stage. Moreover, blood-brain barrier impairment after SAH has been reported to correlate with the development of cerebral vasospasm [22,23,24]. These observations suggest endothelial dysfunction is one of pathological mechanisms in post-SAH vasospasm.

It is well known that NMDAR antagonists may provide neuroprotection to limit secondary neuronal damage in different animal models of neurological disease [15]. Several reports have also proposed that NMDA antagonists can attenuate BBB impairment in SAH and other CNS insults [10,11,20]. In addition, a NMDAR antagonist, remacemide hydrochloride, appears effective against SAH-induced vasospasm [25]. The findings of this study also showed memantine reduced vasospasm after SAH. Because NMDA antagonists are not known to have anti-oxidant properties, the antagonism of NMDARs may be involved in the prevention of cerebral vasospasm. Memantine might reduce endothelial excitotoxicity and result in the improvement of endothelial dysfunction and attenuation of vasospasm.

### 3.2. Memantine Ameliorates Vasospasm via Restoring eNOS Functionality

eNOS is a homodimer that facilitates NO production, a known mediator of vascular tone [8,26]. Phosphorylation of eNOS at Ser1177 is associated with an increase in enzyme eNOS activity [8,26]. Several reports reveal that experimental SAH is associated with increased phosphorylation of Ser1177 in eNOS [8,26,27], but this was paradoxically accompanied by the development of vasospasm. Later, several studies demonstrated that eNOS uncoupling under oxidative stress after SAH facilitates the formation of superoxide anion radical instead of NO [8,26], leading to vasospasm. The phosphorylation of eNOS may suggest an adaptive mechanism against eNOS uncoupling and vasospasm. Several compounds have been demonstrated to re-couple eNOS, restore the phosphorylation of eNOS and finally alleviate the vasospasm after SAH [26,27]. The present study showed that memantine could restore eNOS phosphorylation and attenuate vasospasm after SAH, suggesting a possible effect of memantine on eNOS re-coupling. Further work is required to establish this.

### 3.3. The Medical Management of Delayed Vasospasm

It has been demonstrated that nimodipine, a calcium channel blocker, is the only drug which can reduce the proportion of patients with delayed ischemic neurological disease and poor outcome after SAH [28]. Scientists are still searching for new drug for the prevention of treatment of vasospasm. It has been demonstrated that glutamate-based therapies are beneficial to acute stage of numerous neurological insults, as well as SAH [10,11,15]. The results of this study warrant use of glutamate-based therapies on the prevention of vasospasm. However, the pathological mechanism of delayed vasospasm is multifactorial. Until now, no individual agent has yet progressed beyond the experimental phase to prevent its occurrence [29]. Combination therapy may circumvent some limitations of single-drug therapy by extending the therapeutic time window, or by limiting side effects. A large number of studies indicate that inflammation significantly contributes to the pathogenesis of vasospasm [30]. Several anti-inflammatory drugs have shown promise as treatment options for vasospasm with their anti-inflammatory properties [31,32] and also appear effective in SAH and other neurological diseases [33,34]. Therefore, it merits further examination about combinations of these drugs, which are evidenced efficient in their respective therapeutic categories, for possible synergic interactions in treating SAH and vasospasm.

## 4. Experimental Section

### 4.1. Experimental Paradigm

Animal use complied with the National Cheng Kung University Animal Ethics Committee ethical guidelines. The care and handling of the animals were in accordance with the National Institute of Health guidelines for ethical animal treatment. Male Sprague-Dawley rats weighing 350–450 g were randomly divided into three weight-matched groups: (i) Sham (surgery without SAH insult); (ii) SAH + vehicle (SAH + V) group (SAH animals treated with saline); and (iii) SAH + memantine (SAH + M) group (SAH animals treated with memantine).

### 4.2. Experimental SAH Model

An SAH endovascular perforation model was produced as previously described, with slight modifications [12]. After making a 4 cm skin incision over anterior midline neck, the right external and internal carotid artery (ICA) was isolated. A sharpened 3–0 prolene filament (Ethicon, Inc., Taipei, Taiwan) was introduced into the external carotid artery stump, which was ligated and advanced through the cervical ICA into the intracranial ICA until the ipsilateral CBF decreased, as indicated by the Laser Doppler Flowmetry (AD Instruments, Colorado Springs, CO, USA). The filament was advanced 3 mm further to puncture the cerebral ICA at the bifurcation level. Rats in the sham surgery groups underwent these procedures except for the suture perforation. After surgeries, animals were returned to single, warmed cages for self-extubation when rats recovered from anesthesia. Then each rat was taken care of for 1 or 3 days depending on the protocols of experiments.

### 4.3. Drug

Memantine (Sigma Aldrich, Saint Louis, MO, USA) was given intra-peritoneally at a dose of 20 mg/kg body weight immediately after the insult, and then twice daily at a dose of 1 mg/kg until the animals were euthanized. The sham group received equal volumes of saline via intra-peritoneal injection. All evaluations after surgeries were performed by investigators blinded to the groupings.

### 4.4. Western Blotting

The ipsilateral hemisphere brain was harvested after sham or SAH surgery. For analysis of the expressions of different proteins, tissue was harvested 24 h after surgery. Western blots were performed as described [35]. Briefly, extracts from the cerebral cortex were prepared by homogenization of the tissues in RIPA lysis buffer (50 mM Tris-HCl, 150 mM NaCl, 1% Igepal CA-630, 2 mM EDTA, 1 mM Na_3_VO_4_, 20 g/mL pepstatin A, 20 g/mL leupeptin, 20 g/mL aprotinin, 1 mM PMSF, and 50 mM NaF). The extracts were then analyzed by 8%–12% SDSPAGE. After transfer to nitrocellulose membranes, the blots were blocked with 5% non-fat dry milk in PBST (PBS plus 0.1% Triton X-100) for 1 h followed by blot hybridization with one of the following primary antibodies: anti-phosphorylated S1177-eNOS (1:1000, Cell Signaling, Danvers, MA, USA) and anti β-actin (1:20,000, Sigma, Gillingham, UK) overnight at 4 °C. The membranes were washed thrice for 10 min each in PBST, and then incubated with anti-rabbit IgG (1:5000, Gene Tex, Irvine, CA, USA) conjugated to horseradish peroxidase for 1 h. The membranes were then washed thrice for 10 min each and protein expression was visualized by an enhanced chemi-luminescence kit (Pekin Elmer, Waltham, MA, USA) and exposure to film. The relative band intensities were normalized against that of actin and expressed as means ± standard error of mean (SEM).

### 4.5. Hematoxylin and Eosin Staining and Measurement of Basilar Artery (BA) Cross Section Area

Cerebral vasospasm was evaluated at 72 h after SAH. The rats were anesthetized and perfused trans-cardially in PBS with 4% paraformaldehyde. The brains were removed and immersed overnight in 4% paraformaldehyde solution with 20% sucrose. The brain regions were examined via coronal section (12 mm thick) through the BA at the same point, about two thirds of the distance from the proximal side to avoid arterial branches. For each animal, 4–5 consecutive sections were selected for further staining. The sections then were transferred to 3,3ʹ-diaminobenzidine (DAB) incubation medium with hydrogen peroxide. After visualization of the reaction product, sections were washed in 0.1 M phosphate buffer, stained with hematoxylin, and cover-slipped with xylene-based mounting medium. The BA lumen was outlined using the free hand tool to obtain lumen area, which was measured by the digital image system and HistoQuest software provided by TissueGnostics GmbH (Vienna, Austria). The average of all sections from each animal represented as the value of the animal.

### 4.6. Statistical Analysis

All values were expressed as mean ± standard error of mean (SEM). All data were analyzed by means of one-way analysis of variance with Bonferroni *post hoc* test. Statistical significance was set at *p* < 0.05.

## 5. Conclusions

Despite more aggressive treatment strategies, there is still not effective treatment for delayed vasospasm after SAH. Memantine, with its clinical safety, significantly restores eNOS functionality to prevent the occurrence of vasospasm. These findings emphasize its potential for clinical treatment of SAH.
